# Primary Nasopharyngeal non-Hodgkin lymphomas: a retrospective review of 26 Moroccan patients

**DOI:** 10.1186/1472-6815-9-11

**Published:** 2009-11-17

**Authors:** Wafa Allam, Nabil Ismaili, Sanaa Elmajjaoui, Bel K Elgueddari, Mohammed Ismaili, Hassan Errihani

**Affiliations:** 1 Medical Oncology Unit, National Institute of Oncology, University Center, Avenue Allal Alfassi, Alirfane Rabat, Morocco; 2 Department of Radiotherapy, National Institute of Oncology, University Center, Avenue Allal Alfassi, Alirfane Rabat, Morocco; 3 Department of Microbiology, Moulay Ismail University, Meknes, Morocco

## Abstract

**Background:**

Nasopharyngeal non-Hodgkin lymphomas (NNHL) are extremely rare. In this study, we will report the progress achieved in the management of this disease in our institute.

**Methods:**

We retrospectively reviewed the records of 26 patients having primary NNHL who were managed between January 1997 and December 2008, to evaluate and compare their clinical characteristics and treatment outcome. Clinical variables, including age, sex, stage, and treatment modality, were assessed. Disease free survival and overall survival were measured. Survival curves were constructed using the KaplanMeier method. The log-rank test was used to compare them.

**Results:**

Median age of our patients was 52.7 years. Nasal obstruction, nasal discharge and epistaxis were the frequent symptoms in NNHL patients. Histology of NNHL were mainly large B-cell and follicular lymphoma. Four patients (15.4%) were at stage I, 15 (57.6%) at stage II, and 7 (27%) were at stage III/IV. The patients were treated with chemotherapy alone (27%) or chemotherapy plus radiotherapy (73%). At early stage (stage I/II), the patients were managed with chemo-radiotherapy. When the whole treatment was completed, 18 patients (69.2%) achieved complete response and remained disease free. After 25.9 months median follow-up, overall survival at 1 year was 87% and disease free survival at 1 year was 71%. The difference in term of overall and disease free survival between stage I, II, III and IV was significant (Log rank test: p = 0.02 for overall survival and p = 0.01 for disease free survival).

**Conclusion:**

From our study, we conclude that histological characteristics, principle of treatment and outcome of primary NNHL patients are particular and more studies have to be directed.

## Background

Lymphomas represent the second malignant head and neck tumours region after squamous cell carcinoma[1]. It represents 2.5% of head and neck tumours. Oral and para-oral regions constitute the second most affected localisations by extra nodal lymphomas after that of the gastrointestinal tract[2].

NHL lymphomas are mainly diagnosed as nodal involvement. However, they have the particularity to develop, in 40% of the cases, as extra nodal sites[3].

The aim of our work is to report clinical variables, histological, radiological, biological and therapeutical characteristics of our patients with non hodgkin's nasopharyngeal lymphoma at our institution. This study aimed to compare the clinical characteristics and treatment outcome in 26 patients with primary nasopharyngeal non hodgkin lymphoma managed in our institution between1997 and 2008. In addition, we tried to determine the overall survival and disease free survival of our patients.

## Methods

We searched the patient records at the Department of Clinical Oncology, national institute of oncology of Rabat, for the period 1997-2008 for all tumours coded as primary nasopharyngeal lymphoma. Local ethics committee of our institute has approuved this study.

The files of 26 nasopharyngeal lymphoma patients treated at our institution were thoroughly analysed. Four were excluded because of insufficient clinical information. Patient's medical records were retrospectively studied: demography, clinical stage, histological findings, treatment and outcome. Radiological, pathological and surgical reports were reviewed to determine the stage of the disease at time of histological diagnosis by using Ann Arbor classification system. Data about treatment, notably chemotherapy and radiotherapy were investigated. The date of recurrence, and if applicable, the date of death were also considered. All patients were staged according to the Ann Arbor system [4]. The initial evaluation included a complete history and physical examination, endoscopy, and computed tomographic (CT) cervical-thoraco abdominopelvic scan. Full blood counts, a coagulation screen, blood chemistry studies, serum lactate dehydrogenase levels and plain chest radiography were performed in all patients. Bone marrow biopsy was performed to evaluate patients who have bone marrow involvement at first diagnosis. Cheson criteria were used to evaluate the response in all patients[5,6].

The chemotherapeutic regimen mostly used was CHOP 21 (Cyclophosphamide 750 mg/m^2 ^Intraveinously (IV) at day1, doxorubicin 50 mg/m^2 ^IV at day1, vincristine 1,4 mg/m^2 ^with a maximum total dose of 2 mg IV at day1 and prednisone 40 mg/m^2^/day per os from day 1 to 5). Radiotherapy (RT) was administered to cover the primary tumour and all sites of potential contiguous spread, using a cobalt-60 source in the Waldeyer Ring and in the cervical lymph nodes areas by 3 fields: two opposed lateral fields and one anterior. The total dose of RT was 40 Gy delivered in 2 Gy daily fractions, 5 days per week.

### Statistical analysis

Descriptive statistics with 95% confidence interval (CI) were calculated according to standard procedure. A complete response (CR) was defined as the complete disappearance of all evidence of disease. A partial response (PR) was defined as a reduction of at least 50% in the initial tumour volume without the appearance of new lesions. Disease-free survival (DFS) was measured from the date of complete response to the date of first relapse. Overall survival (OS) was measured from the date of diagnosis to the date of death or last follow-up. Survival curves were constructed using the KaplanMeier method. Patients who failed to achieve complete remission were considered to have relapsed at time zero when constructing the DFS. The log-rank test was used to compare survival curves. All tests were 2-tailed and conducted at a 5% significance level.

## Results

Sex ratio was equal to 1.5:1 with a female predominance; median age was of 52.7 years ranging between 31 and 87 years. 26.9% of the patients had a history of tobacco use. The most frquent symptoms at first diagnosis were: nasal obstruction (88.6%), hypoacousia (88.4%), epistaxis (33.3%), hearing impairment (19,23%) and rhinorrhea (15.3%). 27% of the patients (7 patients) have systemic symptoms (Fever, shudder, sweat, and weight loss more than 5 kg/month). Physical exam showed that clinical cervical lymph nodes were bilateral in 65.3% of patients and unilateral in 19.2%.

### Histology

As shown in Table 1, a variety of histological subtypes were identified in NNHL, using the WHO classification. Histological analysis showed follicular lymphoma in 7 cases (26.9%), large B-cell lymphoma in 13 cases (50%), T-cell lymphoma in 4 cases (15.3%), diffuse mixed B-cell (3,84%), and extranodal marginal zone B-cell lymphoma of mucosa-associated lymphoid tissue type malt (3,84%) [Table 1]. Staging work up was composed of computed tomography scan of the head and neck, lung, abdomen, pelvis, and of bone marrow biopsy. Full blood counts, a coagulation screen, blood chemistry studies, serum lactate dehydrogenase levels were achieved. According to the Ann Arbor classification, four patients (15.4%) were at early stage I, 15 (57.6%) were at stage II, and 3 (11,53%) were at stage III and 4 (15,38%) at stage IV.

**Table 1:** Histological classification and immunophenotype of 26 patients with primary non hodgkin nasopharyngeal lymphomas

	Non hodgkin nasopharyngeal lymphoma (n = 26) (%)
Follicular grade 2	5 (19,23)
Follicular grade 3	2 (7,69)
T cell	4 (15,38)
Diffuse, large B cell	13 (50)
Diffuse, mixed, small and large cell, B	1 (3,84)
Malt	1 (3,84)

### Therapy

Treatment consisted of the combination of chemotherapy and radiotherapy if localised disease (stage I and II) and of only chemotherapy if advanced disease (stage III and IV). The patients that were at stage III and who achieved partial response according to Cheson criteria (up to 50% after the end of chemotherapy treatment), received complementary treatment with radiotherapy in residual sites in attempt to achieve complete response. Although a minimum of six courses was planned for all patients who required chemotherapy, the number given varied according to the tumour response and patient tolerance. A median number of 5 (range 2-8) courses was given to NNHL patients. 73% of patients were managed by 4 to 6 cycles of CHOP21 chemotherapy followed by radiotherapy. None received prophylactic intrathecal chemotherapy.

Before radiotherapy, all patients received dental care. Radiological, clinical, and biological assessment of the disease was performed after 3, 6 and 8 cycles of chemotherapy.

### Response

At the end of total treatment, 18 patients (69.2%) achieved complete response and remained disease free, while 4 (15.4%) achieved partial response. However, for four patients (15.4%) the disease was progressing. In this case also, the 4 patients received a second line chemotherapy. Local relapse occured in two patients after 6 and 13 months. None of the patients had recurrence in the central nervous system, despite its close proximity to the primary tumour.

### Survival

**Figure 1:**
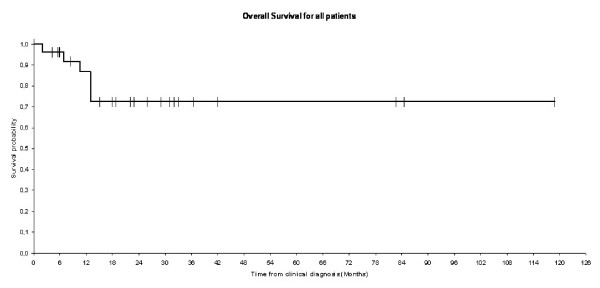
**Kaplan-Meier estimates for overall survival for the entire patients**.

**Figure 2:**
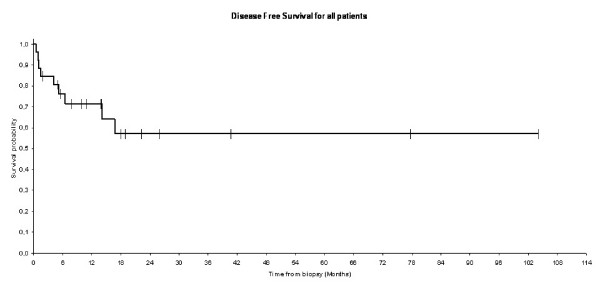
**Kaplan-Meier estimates for disease free survival for the entire patients**.

**Figure 3:**
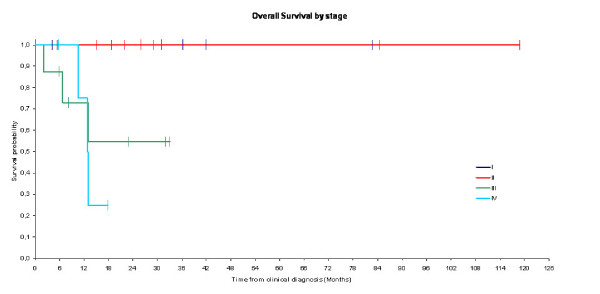
**Kaplan -Meier curves for the Overall survival by stage (Log-rank test: p = 0.02)**. No difference between the curves for stage I and II: (curves are superimposed), but a significant difference with advanced stages III and IV.

**Figure 4:**
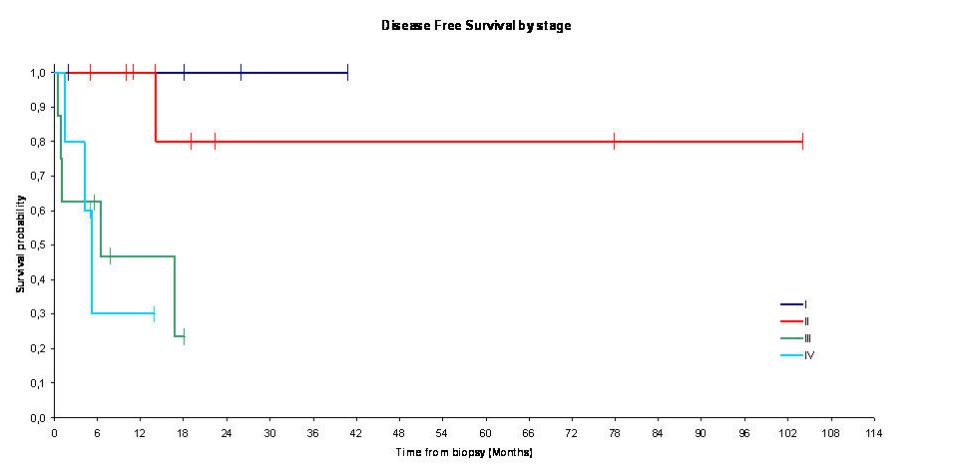
**Kaplan-Meier curves for disease free survival according to the NNHL stage**. (Significant difference between the stages: Log-rank test: p = 0.01)

After a median follow-up of 25.9 months (range 2-62), 19 patients were disease free up to their last follow-up and 5 patients did not return for follow up examinations. Median overall survival at 1 year was equal to 87% (Figure 1). After 18.1 months follow-up, disease free survival rate at 1 year was equal to 71% (Figure 2). The difference in term of overall survival and disease free survival between stage I, II, and III/IV was statistically significant (Log rank test: P = 0.02 for survival and P = 0.01 for progression free survival) (figures 3 and 4). Any episode of grade 3-4 toxicities neitehr of febrile neutropenia was noted. A radiotherapy's toxicity (a grade 3 stomatitis) was registred in one patient, and 30% of patients that received radiotherapy had xerostomia.

Large B cell subgroup: among this 26 patients, 50% were large B-Cell lymphoma with a median age of 65,12 years, and a sex ratio in favor of female sex (2:1). 50% were stage II, 37,5% stage IV and 12,5% stage I. 37,5% had both sequential chemo and radiotherapy, and 62,5 had only chemotherapy because of spread disease. Objective response rate was about 87,5%(whatever the stage). Survival rate was 75% at 2 years.

## Discussion

Patients having NHL represent a heterogeneous group of malignant proliferation of the lymphoid system. The origin of the disease was extra-medullary. This heterogeneity induces different clinical, pathological, immunological and cytogenetical presentations. Therefore, the prognosis is different between the different forms. The incidence of NHL increased continuously since 1950, particularly the large B-cell lymphoma sub-type because of HIV epidemy. Despite the close anatomical relationship between the various structures in the head and neck region, non-Hodgkin's lymphomas arising in these areas are a diverse group of diseases and may have a different natural history and response to therapy.

The NHL lymphomas represent the majority of head and neck lymphomas (65-70%)[7]. The *Waldeyer ring *was the site most frequently affected by NHL (ie, the tonsils, nasopharynx and the base of the tongue)[8]. Few cases of primary NHL of the nasopharynx were reported in the literature, and the series published up to now give incomplete information on NHL evolution. A number of authors investigated Head and neck lymphomas and demonstrated a large predominance of diffuse large B-cell lymphoma sub type as shown in our study[8-10]

Median age in our series was 52,7 years, most lower than that reported by Kemp et al[8]. This author reported a median age of 71 years, which was approximately similar as reported in others series[10-13]. In our series, 57.9% of patients were of age lower than 50 years.

Most of our patients were female. The sex ratio (male:female) was equal to 1:1.5. A similar sex ratio (0.5:1) was reported by Solomides et al[9]. However, Kemp et al., [8] and de Urquhart et al.[13] didn't found any sex predominance. In contrast, in another series the authors showed male predominance with a sex ratio equal to 1.67:1 (25 males and 15 females)[14].

The most important symptoms of Head and Neck NHL were enlarged masses with or without ulceration and pain. In our series, the symptoms were nasal obstruction (88.6%), hypoacousia (88.4%), epistaxis (33.3%) and rhinorrhea (15.3%). Systemic B symptoms are less commonly seen[14,15] as found in our series (in 27% of the patients).

The present study shows that NNHL is mostly comprised B-cell disease, as in K. Lei findings [16]. In a study of 54 patients with nasopharyngeal lymphomas, Ye et al. found an equal incidence of B-cell (51%) and T-cell (49%) lymphomas in the nasopharynx [16]. Similarly, in the recent study of 113 patients reported by Cheung et al. [17], lymphoma involving the nasal cavity was mostly of NK/T-cell or T-cell lineage, while almost half of the lymphomas involving the nasopharynx were of B-cell lineage.

The management of NNHL low grade NHL at stage I and II is based on radiotherapy[18]. Other studies showed better results which were in favour of the combination of radiotherapy and chemotherapy as seen in our study[19]. Treatment of localised disease (stage I and II not bulky) with activity index less than 2 and normal LDH level is based on CHOP regimen (3 to 4 cycles) plus Rituximab (if CD20 positive in immunochemistry) followed by radiotherapy (35-40 Gy in the initial site). In our series, all patients with early stage NNHL were managed by sequential radio-chemotherapy, as found in litterature. More than 80% of patients may be successfully treated (the sequential treatment induced 82% global survival and 77% survival without disease recurrence). The adverse events are dominated by hyposialia and hypothyroidia in one case of thyroid lymphoma, 3,84% in our series. In the series of patients with stage I and II Head and Neck NHL reported by Bolla et all, the patients were managed either with radiotherapy alone (40-55 Gy), with radiotherapy followed by prophylactic chemotherapy for 6 month, or with chemotherapy followed by whole brain radiotherapy, intrathecal methotrexate chemotherapy and adjuvant chemotherapy. Five years survival was 60% for patient managed with radiotherapy alone vs. 70% for patients managed with radio-chemotherapy (p = 0.9%)[20]. In the case of NNHL at stages III or IV, the treatment was based on combined chemotherapy. Rituximab-CHOP regimen is considered as a standard treatment of advanced stage (Bulky, III and IV with large B-cell lymphoma positive to CD-20 antigen). The prophylactic strategy was based on intrathecal methotrexate and systemic high dose methotrexate (up to 3 g/m^2^), in addition to conventional chemotherapy (CHOP based chemotherapy) is widely used given the high risk of central system nervous affection in case of high grade nasopharynageal lymphoma.

The standard criteria currently used to evaluate the response to treatment were the criteria established by the International Working Group in 1999[5]. These criteria were recently revised by the standardisation of use of PET-FDG (Positron emission tomography by fluorodextroglucose) in the assessment of the treatment of DLBCL (diffuse large B cell lymphoma) and Hodgkin lymphoma[6].

Prognosis of NNHL is better than nasopharyngeal squamous cell carcinoma's[21]. In the present series we showed that prognosis of NNHL depended particularly on the stage of the disease. Prognosis of stage I and II of NNHL is significantly better than that of stage III and VI (The probability of survival at 18 months was equal to 100% for stage I and II vs 55% for stage III and 25% for stage IV, log-rank test: p = 0.02). Other series confirmed our results and showed better prognosis in case of localised disease and low grade NNHL[15]. Five years survival was equal to 83% for low grade NHL vs 52% for high grade NHL, and was equal to 83% for stages I vs 42% for stage II[22]. Epstein et al reported overall survival of 51.25% and a median survival of de 3.7 years for men and 6.2 years for women[14]. In our series, median overall survival was 87% at one year and disease free survival was 71% at one year after 25.9 months median follow up. In our series, survival rate in DLBCL is 75% at two years and it depends on the stage. Treatement of these patients was similar than litterature, and even the non-use of rituximab (non available in our institute in the Nineties), response rate was satisfactory (87,5%).

## Conclusion

Nasopharyngeal non-Hodgkin lymphomas are rare. In conclusion, lymphomas arising from the nasopharynx appear to be a particular biological entity. NNHL demonstrated a higher frequency of B-cell tumours. Novel strategies in the improvement of prognosis should be addressed in future studies by assessing prognosis factors implicated in studies. From our results, it could be argued that we conclude that histological characteristics, principle of treatment and outcome of moroccan primary NNHL patients are similar in the present study and in litterature and can suggest that survival is highly better in early stages than advanced ones but have to be confirmed.

## Competing interests

The authors declare that they have no competing interests.

## Authors' contributions

WA carried out the conception and design of the study, made the analysis and the interpretation of data, reviewed literature, and drafted the manuscript and revised it critically for important intellectual content. NI carried out the statistical analysis, and the literature review, revised the manuscript critically for important intellectual content. SE participated in the literature review. BeG revised the manuscript critically for important intellectual content. MI helped in drafting the manuscript. HE carried out the conception and design of the study, revised it critically for important intellectual content. All authors read and approved the final manuscript.

## Pre-publication history

The pre-publication history for this paper can be accessed here:

http://www.biomedcentral.com/1472-6815/9/11/prepub
